# mTOR Inhibition Induces EGFR Feedback Activation in Association with Its Resistance to Human Pancreatic Cancer

**DOI:** 10.3390/ijms16023267

**Published:** 2015-02-03

**Authors:** Feng Wei, Yandong Zhang, Li Geng, Ping Zhang, Guangyi Wang, Yan Liu

**Affiliations:** 1 Department of Hepatobiliary and Pancreas Surgery, the First Hospital, Jilin University, Changchun 130021, China; E-Mails: wei_feng@jlu.edu.cn (F.W.); zhangyd78@126.com (Y.Z.); azhangpinga@126.com (P.Z.); 2 Department of General Surgery, the Second Hospital of Jilin University, Changchun 130041, China; E-Mail: gengli79@gmail.com; 3 Genetic Engineering Laboratory of PLA, the Eleventh Institute of Academy of Military Medical Sciences of PLA, Changchun 130122, China

**Keywords:** mTORC1/2 kinase, cell resistance, EGFR signaling, FoxO1/3a, feedback activation, pancreatic cancer

## Abstract

The mammalian target of rapamycin (mTOR) is dysregulated in diverse cancers and contributes to tumor progression and drug resistance. The first generation of mTOR inhibitors have failed to show clinical efficiency in treating pancreatic cancers due in part to the feedback relief of the insulin-like growth factor-1 receptor (IGF-1R)-AKT signaling pathway. The second generation of mTOR inhibitors, such as AZD8055, could inhibit AKT activation upon mTOR complex 2 (mTORC2) inhibition. However, whether this generation of mTOR inhibitors can obtain satisfactory activities in pancreatic cancer therapy remains unclear. In this study, we found AZD8055 did not show great improvement compared with everolimus, AZD8055 induced a temporal inhibition of AKT kinase activities and AKT was then rephosphorylated. Additionally, we found that AZD8055-induced transient AKT inhibition increased the expression and activation of epidermal growth factor receptor (EGFR) by releasing its transcriptional factors Fork-head box O 1/3a (FoxO1/3a), which might contribute to cell resistance to AZD8055. The *in vitro* and *in vivo* experiments further indicated the combination of AZD8055 and erlotinib synergistically inhibited the mTORC1/C2 signaling pathway, EGFR/AKT feedback activation, and cell growth, as well as suppressed the progression of pancreatic cancer in a xenograft model. This study provides a rationale and strategy for overcoming AZD8055 resistance by a combined treatment with the EGFR inhibitor erlotinib in pancreatic cancer therapy.

## 1. Introduction

Pancreatic cancer, which is the fourth leading cause of cancer-related deaths worldwide, is often associated with very poor prognosis because of the limitations of surgery, the modest response and the subsequent resistance based on either conventional chemotherapy or radiotherapy [1,2]. Although numerous advances in understanding the molecular biology of pancreatic cancer and in diagnosis and treatment regarding *KRAS* mutations, tumor metabolism, and tumor immunology have been made, minimal progress has been achieved in improving the survival of patients [3,4].

The mammalian target of rapamycin (mTOR), which is a central regulator of cell growth and cell apoptosis, contributes to tumor progression and drug resistance [5]. We and others have previously reported that targeting the mTOR signaling pathway might provide novel therapeutics for clinical pancreatic cancer treatment [6,7]. However, the first generation of mTOR inhibitors failed to obtain satisfactory clinical activities, primarily due to the induction of AKT phosphorylation because of the relief of insulin-like growth factor-1 receptor (IGF-1R) signaling pathway feedback [8,9]. In response to this problem, the second generation of mTOR complex 1/complex 2 (mTORC1/C2) dual inhibitors have been developed. AZD8055, which is an adenosine 5'-triphosphate (ATP)-competitive inhibitor, induces not only better mTORC1 inhibition than rapamycin but also a significant decrease in AKT phosphorylation upon mTORC2 inhibition [10,11]. AZD8055 has been shown to inhibit cell proliferation in several solid tumors [12,13] and to sensitize tumor cells to chemotherapies [14,15,16]; however, AZD8055 could also initiate the unexpected activation of phosphatidylinositol 3-kinase (PI3K)/AKT and of certain receptor tyrosine kinases (RTKs), such as HER3 or IGF-1R, in breast cancer or non-small cell lung cancer (NSCLC) cells [17,18].

Epidermal growth factor receptor (EGFR) belongs to the RTK protein family and is dysregulated in the majority of malignant tumors, such as lung cancer, colorectal carcinoma, breast and head/neck cancers [19,20]. The aberrant activation of EGFR leads to the triggering of downstream signaling cascades, including the Ras/Raf/MEK/ERK, PI3K-AKT and JAK/STAT pathways, which contribute to tumor progression, metastasis and therapeutic resistance [21,22]. Erlotinib is a low-molecular-weight inhibitor of EGFR and exhibits >100-fold selectivity for EGFR over other RTKs [23].

In this study, we discovered that AZD8055 failed to induce robust and persistent cell growth inhibition of pancreatic cancer cells. Although AZD8055 clearly inhibited both mTORC1/C2 and AKT activation, AKT inhibition was transient. Intriguingly, we found that the increase in EGFR expression paralleled the AKT inhibition, which suggested the possibility that AKT inactivation is associated with EGFR up-regulation. Through further exploration, we found that AZD8055 induced the temporal inhibition of AKT by releasing the activity of Fork-head box O (FoxO), leading to the transcriptional increase in EGFR expression. Then, the EGFR-dependent activation of AKT and other downstream substrates, such as ERK, might contribute to cell resistance to AZD8055. Finally, we confirmed that the inhibition of EGFR by erlotinib significantly sensitizes pancreatic cancer cells to AZD8055 *in vivo* and *in vitro*, which might suggest a novel strategy for pancreatic cancer therapy.

## 2. Results and Discussion

### 2.1. AZD8055 Transiently Inhibits Pancreatic Cancer Cell Growth

**Figure 1:**
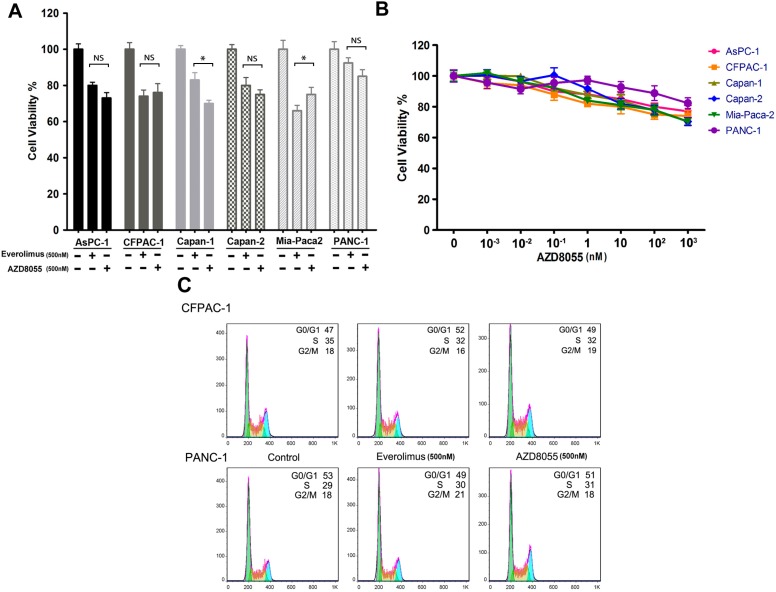

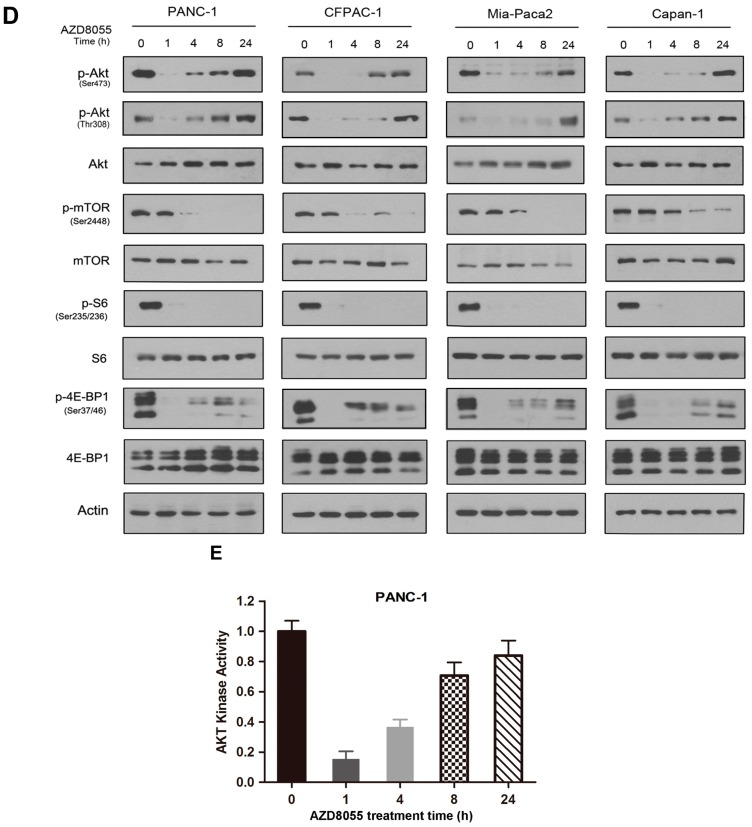
AZD8055 transiently inhibits cell growth of pancreatic cancer. (**A**) The pancreatic cancer cells were treated with AZD8055 or everolimus for 72 h and subjected to cell viability assay. Error bars represent as mean ± SD. NS, not significant; *, *p* < 0.05; (**B**) cells were treated with AZD8055 in the indicated concentrations and subjected to cell viability assay. Error bars represent as mean ± SD.; (**C**) CFPAC-1 and PANC-1 cells were treated with AZD8055 or everolimus for 24 h and cell cycle were analyzed by flow cytometry. The different colors under the curves were used to describe the cells distributed in the different phases of cell cycle more vividly; (**D**) cells were treated with AZD8055 for the indicated hours and examined by western-blot; (**E**) PANC-1 cells were treated with AZD8055 for the indicated hours and AKT kinase activities were examined by *in vitro* AKT kinase assay. Data are representative of three experiments.

To evaluate the efficacy of AZD8055 in pancreatic cancer, we compared a panel of six pancreatic cancer cell lines in response to AZD8055 and everolimus. As shown in Figure 1A, no large difference in cell viabilities was observed after treatment for 72 h, and cell viabilities were greater than 60% in all six cell lines. Furthermore, all cells displayed resistance to AZD8055, even when the concentration was increased to 10^3^ nM (Figure 1B). Meanwhile, the results of the flow cytometry assay indicated that neither everolimus nor AZD8055 could effectively arrest the cell cycle at the G0/G1 phase, and slight changes in the disturbance of the cell cycle were found after treatment with AZD8055 or everolimus (Figure 1C).

Numerous studies have disclosed the mechanism of cell resistance to everolimus, which is associated with AKT (S473) feedback activation. In this study, we asked why AZD8055, as an mTORC1/C2 dual inhibitor, also failed to inhibit cell growth. Three pancreatic cancer cell lines were treated with AZD8055 (500 nM), and we found that AKT (S473/T308) phosphorylation paralleled phosphorylation of the mTOR substrates S6 (ribosomal protein S6) and 4E-BP1 (the eukaryotic translation inhibition factor 4E-binding protein 1) within 4 h of treatment and that S6 and 4E-BP1 phosphorylation was then persistently inhibited. However, AKT (S473/T308) phosphorylation began to increase after 4 h and rebounded to normal levels after 24 h of treatment (Figure 1D). To further confirm whether AZD8055-induced rapid inhibition and subsequent reinduction of AKT phosphorylation would influence AKT activity, an *in vitro* AKT kinase assay was performed using PANC-1 cells treated with AZD8055 for up to 24 h. As shown in Figure 1E, AKT kinase activity decreased to the nadir of 15.7% of the untreated level after for 1 h of treatment and then rebounded up to 84% of the baseline after 24 h of treatment. This result is consistent with the change in AKT phosphorylation. Collectively, these data suggested that AZD8055 treatment results in a temporal inhibition of AKT kinase activities in pancreatic cancer cells and that AKT rephosphorylation might contribute to cell resistance to AZD8055.

### 2.2. AZD8055 Induces Epidermal Growth Factor Receptor (EGFR) Up-Regulation in an AKT-Dependent Manner in Pancreatic Cancer Cells

**Figure 2:**
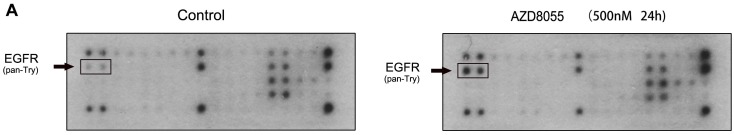

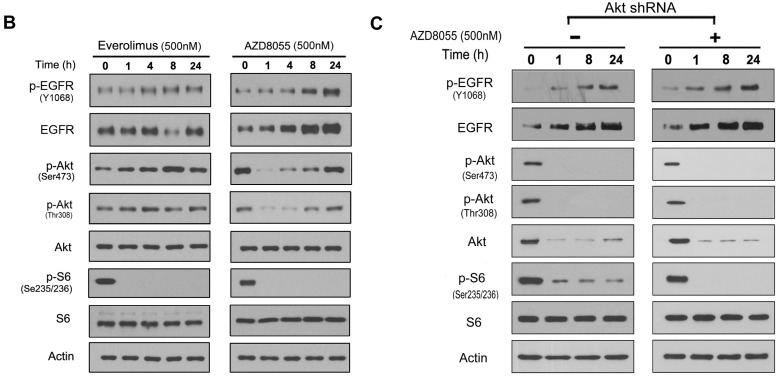
AZD8055 induces EGFR upregulation in an AKT dependent manner. (**A**) PANC-1 cells were untreated or treated with AZD8055 and lysates were applied to phospho-RTK array. The p-EGFR dot blots were indicated by arrow; (**B**) PANC-1 cells were treated with AZD8055 for the indicated hours and EGFR (T/P), AKT (T/P) and ribosomal protein S6 (S6) (T/P) proteins were examined by western-blot; (**C**) PANC-1 cells were transfected with AKT shRNA and treated with AZD8055 for the indicated hours, then above proteins were examined by western-blot.

The above studies indicated that AZD8055 induces the rephosphorylation of AKT; however, the underlying mechanism is not fully understood. AKT is the downstream kinase of receptor tyrosine kinase signaling pathways, and PI3K inhibition has been reported to relieve the feedback suppression of HER family members in breast cancer [24,25]. Therefore, we hypothesized that AZD8055-induced AKT transient inhibition leads to the activation of certain RTKs in pancreatic cancer cells, contributing to AKT rephosphorylation. First, an anti-phosphotyrosine receptor antibody array was performed to assess the RTK phosphorylation levels induced after exposing PANC-1 cells to AZD8055 (500 nM) for 24 h. As shown in Figure 2A, EGFR phosphorylation was greatly induced, and no change was observed for other RTKs, such as HER2, fibroblast growth factor (FGF) and hepatocyte growth factor (HGF). Then, Western Blot analysis showed results similar to the observations above. Interestingly, AZD8055, but not everolimus, induced EGFR over-expression and activation in association with the transient inhibition of AKT (S473/T308) after treatment for 1 to 24 h (Figure 2B). These data indicated that AZD8055 specifically induced the feedback activation of EGFR and might be associated with transient AKT inhibition.

To further confirm whether AKT inhibition plays a key role in mediating EGFR up-regulation in AZD8055-treated cells, we inhibited AKT kinase using a specific AKT shRNA in PANC-1 cells. As shown in Figure 2C, EGFR was upregulated in AKT shRNA-transfected cells with or without AZD8055 treatment. These data suggested that AKT inhibition is necessary for EGFR feedback activation in pancreatic cancer cells. Taken together, these data indicated that AZD8055 induced EGFR up-regulation through an AKT-dependent pathway and then activated the EGFR and AKT signaling pathways, which might contribute to cell resistance to AZD8055 in pancreatic cancers.

### 2.3. AKT Inhibition Releases Fork-Head Box O (FoxO), Contributing to AZD8055-Induced EGFR Up-Regulation

**Figure 3:**
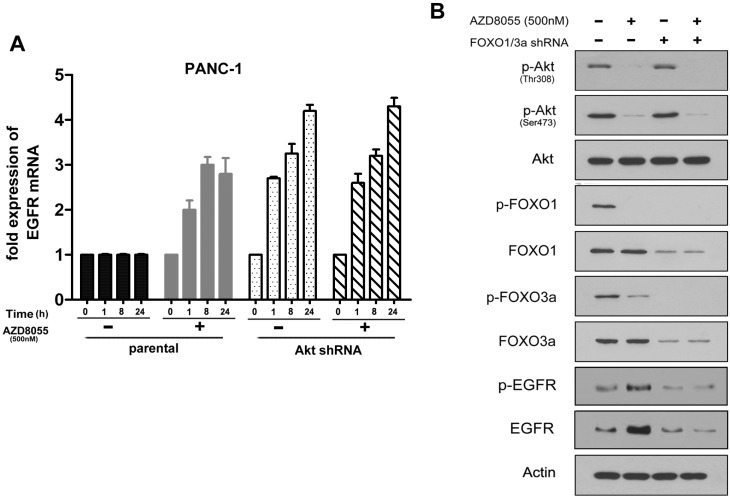
AKT inhibition induces EGFR upregulation in mRNA level in a Fork-head box O (FoxO)-dependent manner. (**A**) PANC-1 cells were transfected with AKT shRNA and treated with AZD8055 for indicated hours, then relative fold change of EGFR mRNA were analyzed by RT-PCR. GAPDH was used as a blank control. Error bars represent as mean ± SD.; (**B**) PANC-1 cells were transfected with FoxO1/FoxO3a siRNAs and treated with AZD8055 for 1 h, then AKT (T/P), FoxO1 (T/P), FoxO3a (T/P) and EGFR (T/P) proteins were examined by western-blot.

To further elucidate the mechanism of AZD8055-induced EGFR upregulation, real-time PCR was performed to examine the mRNA level of EGFR after the exposure of parental and AKT shRNA-transfected PANC-1 cells to AZD8055. As observed in Figure 3A, the EGFR mRNA level was induced above 4-fold in AKT shRNA-transfected cells with or without AZD8055 treatment for 24 h. In contrast, AZD8055 treatment is necessary for EGFR induction in parental cells, resulting in approximately a 3-fold increase. Intriguingly, the mRNA level of EGFR continued to increase from 1 to 24 h in AKT shRNA-transfected cells; however, the mRNA level of EGFR in parental cells reached a maximum at 8 h and then began to decline after exposure to AZD8055. This difference could be explained that specific shRNA-induced AKT inhibition is stronger and more persistent than AZD8055. This result suggested that AZD8055 induces EGFR overexpression at the mRNA level and that this induction is AKT inhibition-dependent.

In the absence of stimuli, Fork-head box O 1/3a (FoxO1/3a), which are the downstream effectors of the PI3K/AKT signaling pathway, reside in the nucleus in their dephosphorylated forms and bind to DNA or other transcription factors to regulate diverse genes involved in cell survival and apoptosis [26]. AKT causes the phosphorylation (inactivation) of FoxO1/3a and induces their translocation from the nucleus to the cytoplasm; thus, phosphorylated FoxO1/3a failed to regulate their target genes, including the RTKs [27]. To clarify whether FoxO1/3a contribute to AZD8055-induced EGFR upregulation, PANC-1 cells were exposed to AZD8055. We found that p-FoxO1/3a strongly decreased in parallel with the reduction of AKT phosphorylation and that EGFR expression simultaneously increased (Figure 3B). These data suggested that FoxO1/3a might be the key mediators of EGFR expression downstream of AKT after AZD8055 treatment. To verify this idea, FoxO1/3a were depleted in combination using their specific siRNAs, and the effect on the level of EGFR expression was examined (Figure 3B). We found that the combined inhibition of FoxO1/3a potently diminished EGFR induction. All of these data indicated that FoxO1/3a activation in response to AZD8055-induced AKT inhibition is required for the up-regulation of EGFR in pancreatic cancer cells.

### 2.4. Combined Inhibition of the Mammalian Target of Rapamycin (mTOR) and EGFR Persistently Inhibits AKT Activation and Synergistically Inhibits Cell Growth in Vitro

**Figure 4:**
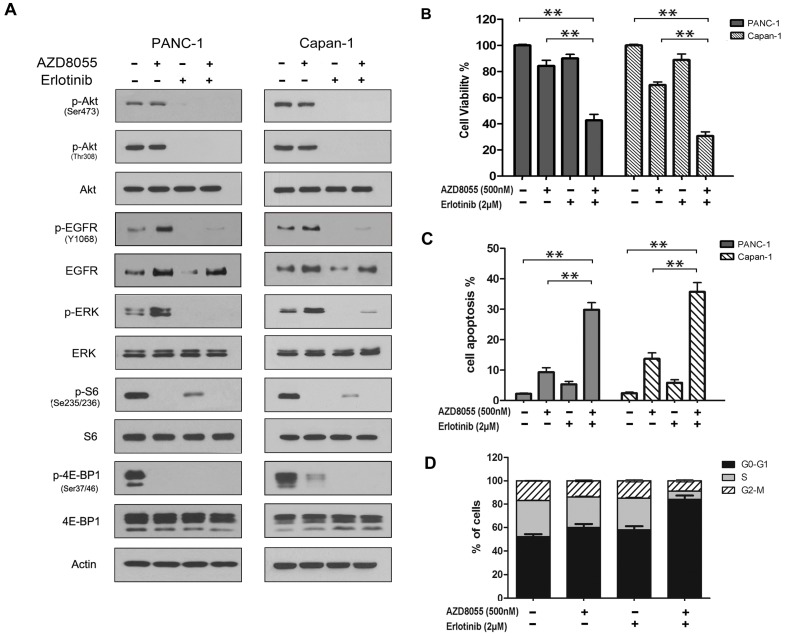
Combined inhibition of the mammalian target of rapamycin (mTOR) and EGFR synergistic inhibit cell growth *in vitro*. (**A**) PANC-1 and Capan-1 cells were treated with AZD8055 and erlotinib, alone or in combination for 24 h, western-blot were used to examine the indicated unphosphorylated or phosphorylated proteins; (**B**) the cells were treated with AZD8055 and erlotinib, alone or in combination for 72 h, then subjected to cell viability assay; (**C**) the cells were treated with AZD8055 and erlotinib, alone or in combination for 24 h, and cell apoptosis were analyzed by flow cytometry; (**D**) the cells were treated with AZD8055 and erlotinib, alone or in combination for 24 h, and cell cycle were analyzed by flow cytometry. Above data are representative of three experiments and the percentage of apoptotic cells were represent as mean ± SD. ** *p* < 0.01 *vs.* control.

The above studies have suggested that EGFR upregulation might contribute to the continuation of mTOR activities in pancreatic cancer cells under AZD8055 treatment. To further clarify this point, PANC-1 and Capan-1 cells were treated with AZD8055, erlotinib or both. Western Blot analysis showed that erlotinib potently inhibited the phosphorylation of EGFR as well as its downstream proteins AKT and ERK and partly inhibited the phosphorylation of S6 or 4E-BP1 [28]. In contrast, the combination of erlotinib and AZD8055 effectively inhibited both mTOR and EGFR, as well as their substrate activities (Figure 4A). Then, the MTT assay showed that erlotinib synergistically enhanced the cell growth inhibition of AZD8055 and that the combination of AZD8055 and erlotinib caused more than 60% cell growth inhibition compared with AZD8055 (less than 30%) or erlotinib (less than 20%) treatment alone (Figure 4B). These data indicated that erlotinib could effectively sensitize pancreatic cancer cells to AZD8055.

We then wondered whether the combination treatment could synergistically arrest the cell cycle and induce cell apoptosis. As observed in Figure 4C, erlotinib or AZD8055 treatment alone had little effect on cell cycle arrest, whereas their combination caused a strong accumulation of cells at the G0/G1 phase (from 52% to 84%) and a significant reduction at the S phase (from 30% to 7%) compared with the control. Additionally, similar data were obtained from Capan-1 cells (data not shown). Then, the Annexin V assay showed that the combination treatment increased cell apoptosis by 29.8% or 35.7% compared with AZD8055 (9.3% or 13.6%) or erlotinib (5.3% or 5.8%) single treatment (Figure 4D), respectively, in PANC-1 and Capan-1 cells. All data indicated that erlotinib sensitized pancreatic cancer cells to AZD8055 at least partly due to the synergistic arrest of the cell cycle and the induction of cell apoptosis.

### 2.5. Combined Erlotinib and AZD8055 Treatment Leads to the Effective Suppression of Pancreatic Cancer Xenografts

**Figure 5:**
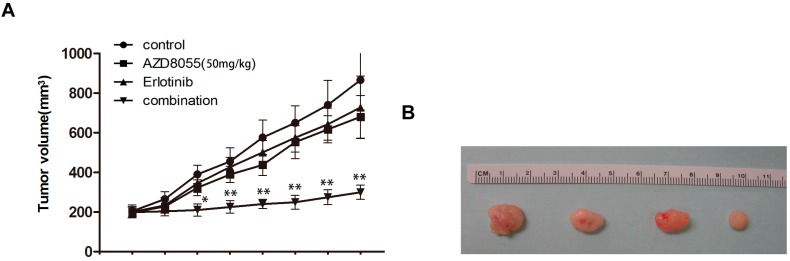

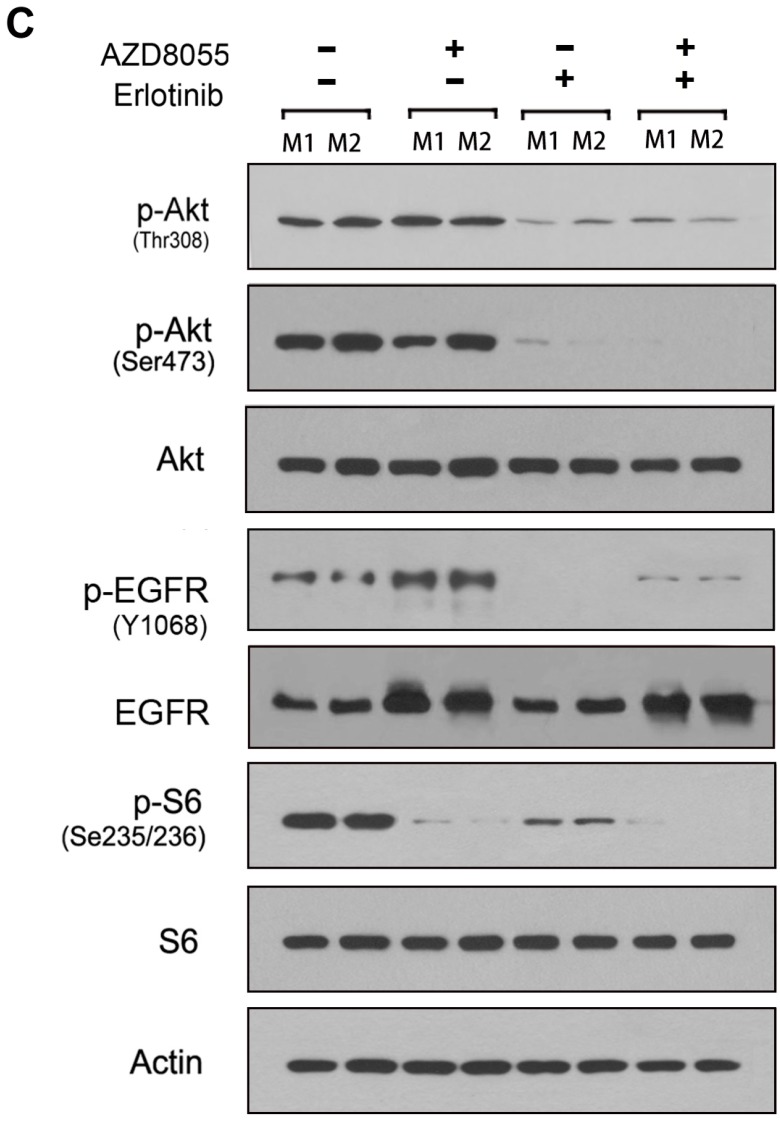
EGFR inhibition improves antitumor efficacy when given in combination with AZD8055. (**A**) The tumor volumes from the same group mice were presented as mean ± SD. and statistically analyzed. *, *p* < 0.05 and **, *p* < 0.01 *vs.* AZD8055 treatment alone; (**B**) At necropsy, the representative xenografts were shown from the left to right with saline, AZD8055, erlotinib, AZD8055 and erlotinib combination; (**C**) the xenografts were lysed and subjected to western-blot for the presence of the indicated proteins in the xenograft tissues.

To further evaluate the therapeutic potential of combined treatment with erlotinib and AZD8055 *in vivo*, mice bearing subcutaneous PANC-1 xenografts were generated and treated as described in the Experimental Section. As indicated in Figure 5A,B, either AZD8055 or erlotinib treatment modestly suppressed tumor growth in pancreatic cancer xenografts. In contrast, a strong antitumor effect was observed when erlotinib was used in combination with AZD8055, which slowed growth and significantly reduced tumor volumes compared with control and single treatment groups. Meanwhile, Western Blot analysis of the freshly removed tumor tissues showed that the combination treatment effectively abolished the activities of both the mTORC1/C2 and EGFR/Akt pathways (Figure 5C). Collectively, all of these results suggested that the combined treatment of AZD8055 and erlotinib synergistically suppressed the xenograft progression of pancreatic cancer in association with mTOR signaling pathway inhibition and EGFR/Akt feedback activation.

## 3. Experimental Section

### 3.1. Materials

AZD8055 was purchased from Selleck Chemicals (Houston, TX, USA). Erlotinib was obtained from LC Laboratories (Woburn, MA, USA). mTOR (T/P), AKT, p-AKT (S473), p-AKT (T308), S6 (T/P), 4E-BP1 (T/P), EGFR (T/P), FOXO1 (T/P), and FOXO3a (T/P) antibodies, as well as FoxO1 and FoxO3a siRNAs, were obtained from Cell Signaling Technology (Beverly, MA, USA). All other reagents were obtained from the stated commercial sources. Human AKT short hairpin RNA (shRNA) and control shRNA plasmids were obtained from Santa Cruz Biotechnology (Santa Cruz, CA, USA).

### 3.2. Cell Lines, Cell Culture and Transfection

All human pancreatic cancer cell lines (PANC-1, AsPC-1, CFPAC-1, Capan-1, Capan-2 and Mia-Paca2) were purchased from the National Rodent Laboratory Animal Resources (Shanghai, China) and conventionally cultured in McCoy’s 5α medium or DMEM medium supplemented with 10% FBS (Invitrogen, Carlsbad, CA, USA). For transient transfection, the cells were seeded in six-well plates overnight, and the specific plasmids were then transfected into cells using Opti-MEM^®^ I with Lipofectamine™ 2000 (Invitrogen) according to the manufacturer’s instructions. The cells were treated or harvested after 48 h of transfection.

### 3.3. 3-(4,5-Dimethylthiazol-2-yl)-2,5-diphenyltetrazolium bromide (MTT) Assay

The cells were seeded in 96-well plates at 6–8 × 10^3^/well, cultured overnight, and then treated with drugs for 72 h at 37 °C. Cell viability was determined using the 3-(4,5-dimethylthiazol-2-yl)-2,5-diphenyltetrazolium bromide (MTT) assay as previously described [29]. Absorbance was measured using a spectrophotometer (Bio-Rad Laboratories, Inc., Hercules, CA, USA) at 490 nm, and cell growth inhibition was calculated using the equation cell viability (%) = (At/Ac) × 100% in which At and Ac represent the absorbance values of treated and control cultures, respectively [9].

### 3.4. Cell Lysate and Western Blot Assay

Total proteins were extracted using cell lysis buffer (Cell Signaling Technology, Beverly, MA, USA) containing protease inhibitor cocktail (Sigma-Aldrich Co., St. Louis, MO, USA). After centrifugation at 15,000 rpm for 15 min, the supernatants were collected and quantified by Bradford assay. Then, equal amounts of protein were subjected to SDS–PAGE and transferred onto nitrocellulose membranes. The membranes were incubated with primary and secondary antibodies and then developed by chemiluminescence [30].

### 3.5. RNA Isolation and Quantitative Real-Time PCR

Total RNA was extracted from cells using TRIzol. In total, 1–10 μg of RNA was reverse transcribed into cDNA using the SuperScript II First-Strand Synthesis System (Invitrogen). Then, aliquots of the reaction mixture were used for PCR amplification with Power SYBR Green PCR Master Mix (Applied Biosystems, Carlsbad, CA, USA). All PCR experiments were performed in triplicate. The primer sequences used are as follows: EGFR sense: 5'-GTGGCTGGTTATGTCCTCATTGCC-3'; EGFR antisense: 5'-ACACTTCTTCACGCAGGTGGCACC-3' [31]; GAPDH sense: 5'-GGTGAAGGTCGGAGTCAACG-3'; and GAPDH antisense: 5'-TGGGTGGAATCATATTGGAACA-3'.

### 3.6. Cell Cycle and Cell Apoptosis Analysis

For cell cycle analysis, the cells were synchronized by serum starvation for 48 h and released into the cell cycle by adding complete medium containing 10% FBS. After being treated with drugs for 24 h, the cells were harvested, fixed with 70% ethanol, and stained with propidium iodide (PI). For cell apoptosis analysis, the cells were treated with drugs for 24 h, and then 1 × 10^6^ cells were collected into each tube, suspended in binding buffer, stained with Annexin V/PI and incubated for 15 min in the dark. Cell cycle and cell apoptosis data were acquired by FACScan (BD Biosciences, Franklin Lakes, NJ, USA) and analyzed by FlowJo software (Tree Star Inc., Ashland, OR, USA).

### 3.7. Phosphorylated Receptor Tyrosine Kinase (RTK) Array

A PathScan Phospho-RTK Signaling Antibody Array Kit (Cell Signaling Technology) was used according to the manufacturer’s instructions. The slide-based antibody array included 28 RTKs. In total, 50 μg of cell lysate was incubated with the block slide overnight, and the membrane was then washed and developed by chemiluminescence.

### 3.8. Animal Studies

The animal studies were performed according to an approved protocol of the Jilin University Institutional Animal Care and Use Committee (Project identification code: 2013-017, Date: 1 March 2013). PANC-1 cells (7 × 10^6^) were resuspended in HBSS and subcutaneously injected into the flank regions of six-week-old nu/nu athymic female mice (approximately 20 g body weight, Shanghai, China). The tumors were allowed to grow to an average volume of 200 mm^3^. Then, the mice were randomly assigned to four groups (*n* = 8): (1) vehicle control (5% DMSO); (2) AZD8055 (50 mg/kg); (3) erlotinib (50 mg/kg); and (4) a combination of AZD8055 (50 mg/kg) and erlotinib (50 mg/kg). The drugs were dissolved in DMSO and administered by oral gavage (0.1 mL/10 g) 3 times per week. The tumor volumes were measured once every 2 days and calculated using the following formula: V = 4/3 × π (length/2 × (width/2)^2^). After 21 days of treatment, the mice were sacrificed and the tumors were removed for subsequent analysis.

### 3.9. Statistical Analysis

All data are presented as the mean value ± standard error. Statistical analysis was performed using GraphPad Prism software (San Diego, CA, USA), as described previously [32]. The statistical significance of differences between two groups was analyzed using the two-tailed unpaired Student’s *t*-test. The results were considered statistically significant at *p* < 0.05.

## 4. Conclusions

Pancreatic cancer therapy remains a great challenge in clinical oncology due to the similar rate between mortality and incidence [33]. Although surgery, radiotherapy and gemcitabine-based chemotherapy could temporarily improve the symptoms, these treatments do not effectively extend patient survival [2,34].

Increasing evidence has indicated that mTOR and its substrates are dysregulated in numerous human carcinomas and that mTOR knockdown by specific siRNAs could inhibit tumor growth in colorectal cancer [35]. Therefore, the mTOR signaling pathway provides a new strategy for cancer therapy. However, the single agent of the first generation of mTOR inhibitors does not have a broad and robust antitumor effect. These inhibitors exert their actions almost exclusively through mTORC1 inhibition; however, the subsequent feedback activation of AKT kinase leads to tumor resistance.

Through better understanding of the mechanisms of mTOR feedback loops, the second generation of mTOR inhibitors, including AZD8055, have been developed. Theoretically, the most important advantages of AZD8055 would be a significant decrease in AKT (S473) phosphorylation upon mTORC2 inhibition and better mTORC1 inhibition, including 4E-BP1, S6K and AKT (T308) phosphorylation. In this study, we confirmed that AZD8055 fails to induce significant cell growth inhibition, and then we demonstrated that AZD8055 causes strong inhibition of 4E-BP1, S6K and AKT S473/T308. However, the inhibition of AKT is transient, occurs quickly and allowing levels to increase again after 8 h. Previous studies have shown that inhibiting the PI3K/AKT pathway can lead to the up-regulation and over-activation of RTKs, such as HER3 (ErbB3), IGF-1R or IR, aimed at maintaining the PI3K pathway in equilibrium in breast cancer or NSCLC [24,36,37]. Considering that AZD8055 could induce AKT inhibition, we found that EGFR, but not other RTKs, could be potently upregulated by AZD8055 in pancreatic cancer cells. Then, we further determined that AZD8055-induced EGFR up-regulation is AKT inhibition-dependent; this result was also confirmed by everolimus-treated cells in which no AKT inhibition and no EGFR upregulation occur.

AKT could phosphorylate the FoxO family of transcription factors, prevent their nuclear translocation, and inhibit their functions [38]. FoxOs were also involved in the induction of the RTKs, such as HER3 and IR, by AKT inhibition [24,27,39,40]. In the present study, we found that AKT inhibition reduced the phosphorylation of FoxO1/3a in association with increased EGFR expression. In addition, we also found that the basal expression of EGFR was maintained at a stable level when FoxO1/3a were inhibited, which indicated that EGFR could also be mediated by some other transcription factors when FoxO1/3a lose their functions with the activated AKT pathway in tumor cells [41].

Our results not only demonstrated that AZD8055 induced the transient inhibition of AKT in parallel with the feedback induction of EGFR, which is associated with cell resistance to AZD8055, but also disclosed the underlying mechanism by which AKT inhibition released the activity of FoxO 1/3a, which mediated EGFR upregulation in pancreatic cancers. Therefore, we proposed that the combination of AZD8055 and EGFR inhibitors would suppress the growth of pancreatic cancer cells with greater efficacy than single treatment because, in addition to AKT kinase, the EGFR pathway also involves many other substrates such as ERK, which is dysregulated in tumor cells and promotes tumor progression [42]. Our studies indicate that erlotinib significantly potentiates the cytotoxic effects of AZD8055 in human pancreatic cancer cells, accompanied by a marked reduction of the S phase of the cell cycle and the induction of cell apoptosis. Consistent with the *in vitro* results, their combination also leads to more effective suppression of pancreatic cancer xenografts than treatment with either therapy alone.

In summary, our study discloses a novel mechanism of AZD8055 induction of pancreatic cancer cell resistance, which is dependent on AKT inhibition-induced EGFR upregulation. Additionally, FoxO1/3a, which are the downstream transcription factors of AKT, are involved in EGFR over-expression. Therefore, the inhibition of EGFR by erlotinib represents an efficient strategy to overcome cell resistance to AZD8055 in pancreatic cancers.
